# Acetylcholinesterase Inhibitory, Antioxidant and Phytochemical Properties of Selected Medicinal Plants of the Lamiaceae Family

**DOI:** 10.3390/molecules19010767

**Published:** 2014-01-09

**Authors:** Sanda Vladimir-Knežević, Biljana Blažeković, Marija Kindl, Jelena Vladić, Agnieszka D. Lower-Nedza, Adelheid H. Brantner

**Affiliations:** 1 Department of Pharmacognosy, Faculty of Pharmacy and Biochemistry, University of Zagreb, Marulićev trg 20, Zagreb 10000, Croatia; E-Mails: svladimir@pharma.hr (S.V.-K.); mkindl@pharma.hr (M.K.); 2 Department of Biotechnology and Pharmaceutical Engineering, Faculty of Technology, University of Novi Sad, Bulevar cara Lazara 1, Novi Sad 21000, Serbia; E-Mail: vladicjelena@gmail.com; 3 Department of Pharmacognosy, Institute of Pharmaceutical Sciences, Karl-Franzens-University Graz, Universitaetsplatz 4, Graz 8010, Austria; E-Mails: agnieszka.ln86@gmail.com (A.D.L.-N.); adelheid.brantner@uni-graz.at (A.H.B.)

**Keywords:** Croatian medicinal plants, Lamiaceae, acetylcholinesterase inhibition, antioxidant, hydroxycinnamic derivatives, rosmarinic acid

## Abstract

The present study aimed to evaluate acetylcholinesterase (AChE) inhibitory and antioxidant activities of Lamiaceae medicinal plants growing wild in Croatia. Using Ellman’s colorimetric assay all tested ethanolic extracts and their hydroxycinnamic acid constituents demonstrated *in vitro* AChE inhibitory properties in a dose dependent manner. The extracts of *Mentha* *x* *piperita*, *M. longifolia*, *Salvia officinalis*, *Satureja montana*, *Teucrium arduini*, *T. chamaedrys*, *T. montanum*, *T. polium* and *Thymus vulgaris* at 1 mg/mL showed strong inhibitory activity against AChE. The antioxidant potential of the investigated Lamiaceae species was assessed by DPPH^•^ scavenging activity and total antioxidant capacity assays, in comparison with hydroxycinnamic acids and trolox. The extracts differed greatly in their total hydroxycinnamic derivatives content, determined spectrophotometrically. Rosmarinic acid was found to be the predominant constituent in most of the investigated medicinal plants (by RP-HPLC) and had a substantial influence on their AChE inhibitory and antioxidant properties, with the exception of *Teucrium* species. These findings indicate that Lamiaceae species are a rich source of various natural AChE inhibitors and antioxidants that could be useful in the prevention and treatment of Alzheimer’s and other related diseases.

## 1. Introduction

Alzheimer’s disease (AD), the most common cause of dementia, is a progressive age-related neurodegenerative disorder which is becoming a serious public health issue and a massive economic burden. AD patients lose their memory, their cognitive abilities and even their personalities may change dramatically. These changes are due to the progressive dysfunction and death of nerve cells responsible for the storage and processing of information [1]. AD arises as a result of malfunction of different biochemical pathways. Multiple pathogenic factors, including aggregated amyloid-β-peptide and tau protein, excessive transition metals, oxidative stress and reduced acetylcholine (ACh) levels have been implicated in AD pathology [2]. Acetylcholinesterase (AChE), the predominant cholinesterase in the brain, hydrolyzes ACh to choline and acetate, thereby terminating the effect of this neurotransmitter at cholinergic synapses. Therefore, AChE is the target of cholinesterase inhibitors used for addressing the cholinergic deficit in AD patients. Despite decades of research and advances in our understanding of its aetiology and pathogenesis, current pharmacotherapeutic options for AD are still very limited and represent an area of need that is currently unmet. The leading AD therapeutics involve AChE inhibitors, resulting in increased acetylcholine concentrations in the synaptic cleft and enhanced cholinergic transmission [3,4]. Compounds showing an AChE inhibitory effect are also used for the treatment of senile dementia, myastenia gravis, Parkinson’s disease and ataxia [5]. Taking into account that the inhibition of AChE has been one of the most used strategies for treating AD and that existing drugs are effective only against mild to moderate type of disease while presenting considerable side effects, the search for new sources of effective and selective antiacetylcholinesterase agents with fewer side effects is imperative. The free radical and oxidative stress theories of aging suggest that oxidative damage is a major player in the degeneration of cells. In this respect, recent findings clearly indicate that oxidative damage is one of the earliest events in the pathogenesis of AD, so targeting oxidative stress could be considered beneficial in both its prevention and treatment [6]. 

Owing to their richness in secondary metabolites that exhibit a remarkable diversity of both chemical structures and biological activities, medicinal plants are being recognized as promising sources of lead compounds for new drugs targeting neurodegenerative diseases [7,8,9]. Croatia is one of Europe’s most biodiversity-rich countries due to its unique geographical position. As an integral part of the Mediterranean area, its flora is represented by a high number of species and subspecies, in total 5,516 taxa belonging to 184 families, among which Lamiaceae could be considered one of the richest in medicinal plants [10]. Lamiaceae species have been reported to possess a wide range of biological activity, and a wide diversity of phytochemicals. Essential oils, hydroxycinnamic acids and flavonoids were found as the major bioactive constituents of the most common Lamiaceae species, such as thyme, rosemary, peppermint, sage, lemon balm and oregano [11]. The essential oil composition of *Thymus vulgaris* can vary greatly and various chemotypes have been recorded, particularly regarding thymol and carvacrol. Other constituents of thyme include rosmarinic acid and flavonoids (luteolin, eriodictyol, apigenin and some methylated flavones) [12,13]. Rosemary leaves contain an essential oil whose composition may vary according to the plant chemotype (eucalyptol, camphor-borneol and α-pinene-verbenone types are distinguished). Rosmarinic acid and flavonoids as well as diterpenes, which are structural derivatives of carnosic acid, belong to the phenolic fraction [14,15]. Menthol and menthone are the major components of peppermint essential oil. The phenolic constituents of peppermint include rosmarinic acid and several flavonoids, primarily eriocitrin, luteolin and hesperidin [16]. The essential oil of *Salvia officinalis* is characterized by a high amount of α- and β-thujone. The non-volatile fraction of sage is mainly composed of various diterpenes, phenolic acids and flavonoids [17,18]. Lemon balm yields only a small quantity of essential oil, with citral and citronellal as the principal components. It also contains hydroxycinnamic acids (rosmarinic, *p*-coumaric and caffeic acids) and flavonoids, e.g., luteolin, quercetin, apigenin and kaempferol [19]. The composition of *Origanum vulgare* essential oil from different geographical origins is most commonly characterised by carvacrol and thymol as the major components, though the proportions vary widely. The phenolic compounds including flavonoids and phenolic acids are another kind of abundant constituent in oregano [20]. Investigation of the species belonging to the genus *Teucrium* revealed the presence of diterpenes, triterpenes, phytosterols, iridoids, flavonoids and essential oils [21,22]. It was found that *Lavandula* species contain essential oil, triterpenes, coumarins, hydroxycinnamic acids and flavonoids [23]. Most of the chemical studies on *Micromeria* and *Calamintha* species were carried out for investigation of their volatile constituents and flavonoids [24,25,26]. 

A large number of species belonging to the genus *Calamintha*, *Lavandula*, *Mentha*, *Melissa*, *Origanum*, *Rosmarinus*, *Salvia*, *Teucrium* and *Thymus* has traditionally been used in Croatia and neighbouring countries to treat respiratory diseases, gastrointestinal problems and various nervous system disorders [27,28,29]. Some of them have been investigated for their antioxidant and neuroprotective effects using various *in vitro* and *in vivo* methods. These activities have mostly been attributed to the presence of polyphenols, particularly rosmarinic acid [30,31,32,33]. Along with other more common hydroxycinnamates, such as caffeic, ferulic, *p*-coumaric and chlorogenic acid, rosmarinic acid is widely distributed in the plant kingdom, occurring particularly in species of the Lamiaceae and Boraginaceae families. Recent biological and pharmacological studies have shown that this phytochemical possesses many beneficial effects, including antiviral, antibacterial, antioxidant, antiinflammatory, antiangiogenic, antidepressive, anticancer and antihepatotoxic activities [11,34,35].

Aiming to discover new and promising sources of potential anti-AD drugs, the present study was undertaken to evaluate the antiacetylcholinesterase and antioxidant properties of selected Croatian medicinal plants belonging to the Lamiaceae family. Additionally, their hydroxycinnamic acid profiles were investigated. 

## 2. Results and Discussion

### 2.1. Acetylcholinesterase (AChE) Inhibitory Activity

Over the past two decades, cholinesterase inhibition has become the most widely employed clinical approach to treating the symptoms of AD. Various plants and phytochemical substances have demonstrated AChE inhibitory activity and thus could be beneficial in the treatment of neurodegenerative disorders such as AD [7]. In this study, the ethanolic extracts of 26 medicinal plants of the Lamiaceae family growing in Croatia were comparatively tested for their inhibitory activity against AChE at concentrations of 0.25, 0.50 and 1 mg/mL by *in vitro* Ellman’s method. To the best of our knowledge, there are no literature data for most of the tested plants concerning the AChE inhibitory properties of their polar extracts, with the exception of *Rosmarinus officinalis* [30,36,37,38,39], *Lavandula angustifolia* [37,38,40], *Salvia officinalis* [40], *Mentha* *x piperita* [36,41], *M. longifolia* [36], *M. pulegium* [39,41], *Melissa officinalis* [37,40,42], *Teucrium polium* [36,42], *Satureja montana* [43] and *Micromeria juliana* [44]. Since Lamiaceae species were found to be rich in phenolic acids as active constituents that significantly contribute to their neuroprotective properties [7], the anti-AChE activities of rosmarinic, caffeic, chlorogenic and ferrulic acid were also examined in this study. The plant alkaloid galanthamine was used as the reference AChE inhibitor. 

**Figure 1:**
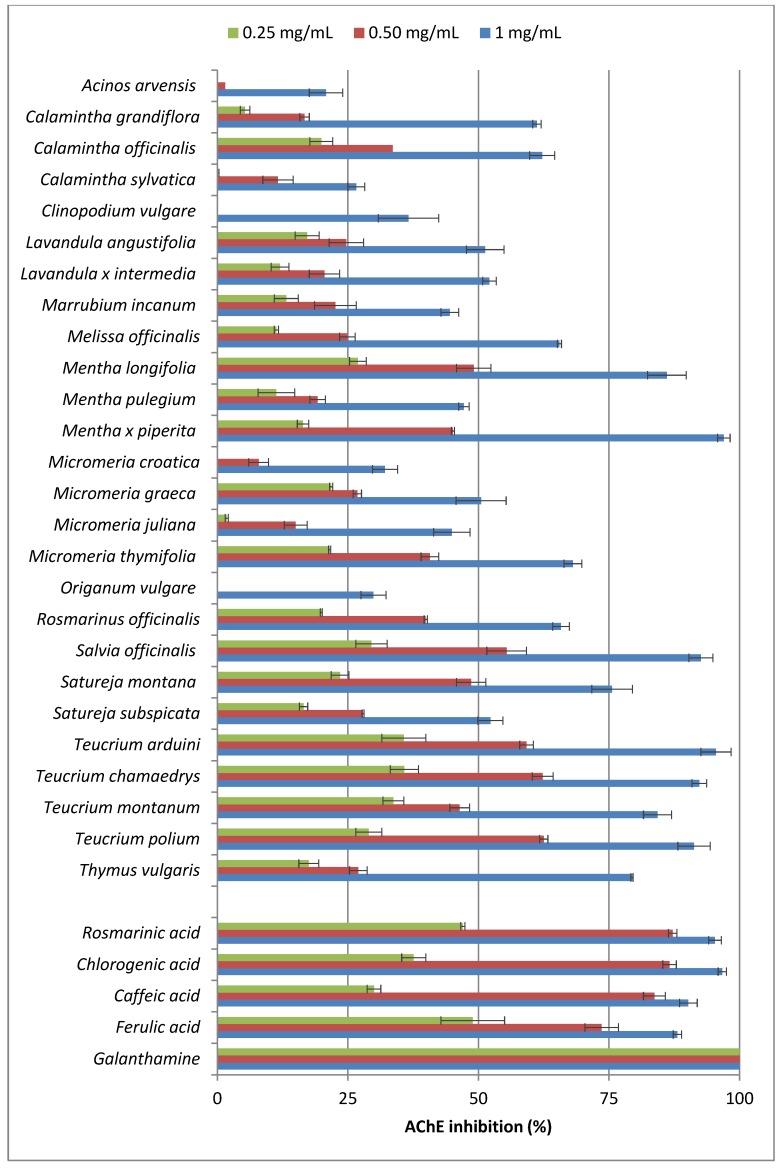
Acetylcholinesterase (AChE) inhibitory activities of the ethanolic extracts of selected Lamiaceae species and their phenolic acid constituents.

All tested ethanolic extracts possessed the ability to inhibit AChE in a dose dependent manner (Figure 1). However, significant differences in AChE inhibitory properties were established between the tested plants, and values of 0%–35.8%, 0%–62.5% and 20.8%–97.0% were obtained for tested concentrations of 0.25, 0.50 and 1 mg/mL, respectively. The most potent plant extracts, with AChE inhibition rates above 75% at 1 mg/mL, were those of *Mentha x piperita*, *M. longifolia*, *Salvia officinalis*, *Satureja montana*, *Teucrium arduini*, *T. chamaedrys*, *T. montanum, T. polium* and *Thymus vulgaris.* Ethanolic extracts (1 mg/mL) of *Calamintha grandiflora*, *C. officinalis*, *Lavandula angustifolia*, *L. x intermedia*, *Melissa officinalis*, *Micromeria graeca*, *M. thymifolia*, *Rosmarinus officinalis* and *Satureja subspicata* demonstrated moderate inhibitory activity (50.5%–68.1%). On the other hand, *Acinos arvensis*, *Calamintha sylvatica*, *Clinopodium vulgare, Marrubium incanum*, *Mentha pulegium*, *Micromeria croatica, M. juliana* and *Origanum vulgare* did not achieve 50% inhibition of enzyme activity.

The tested hydroxycinnamic acids at 0.25 and 0.50 mg/mL inhibited 30.0%–48.9% and 73.6%–86.6% of AChE activity, respectively, which was generally a stronger effect than the tested extracts exerted, though at the highest concentration examined, their AChE inhibition (88.1%–96.7%) was comparable to the activity of the extracts found as the most potent. Galanthamine, used as the reference AChE inhibitor, demonstrated a similar effect at a 1,000 times lower concentration (IC_50_ = 0.122 ± 0.004 μg/mL). These results also confirmed previous findings of several medicinal plants reported to show AChE inhibitory activity, such as *Rosmarinus officinalis* [37,39], *Salvia officinalis* [40], *Lavandula angustifolia* [37,40] and *Teucrium polium* [42]. 

### 2.2. Antioxidant Activity

Since age is a primary risk factor for the majority of AD cases, oxidative stress is inevitably involved in the pathogenesis of AD. Oxidative stress is an imbalance between reactive oxygen species (ROS) production and the intracellular capacity for removing ROS, subsequently leading to excessive damage of DNA, lipids, carbohydrates and proteins in the cell. Neural cells, which suffer functional (ataxia) or sensory loss (dementia) in neurodegenerative diseases, are considered to be more susceptible to oxidative damage as compared to other body tissues. The brain is highly sensitive to oxidative stress as it is rich in peroxidizable fatty acids, has a high oxygen demand and a relative paucity of cellular- and tissue-specific antioxidant mechanisms [6,7]. Antioxidants can delay, inhibit or prevent the oxidation of oxidizable materials by scavenging free radicals and diminishing oxidative stress. The plant kingdom offers a wide range of compounds exhibiting antioxidant activities, especially plant polyphenols that have been investigated with the intent of finding compounds that can protect against a number of diseases related to oxidative stress and free radical-induced damage [45,46].

The free-radical scavenging activities of the ethanolic extracts of selected Lamiaceae species were evaluated by the commonly used DPPH assay, in comparison with pure hydroxycinnamic acids and trolox as a reference antioxidant. DPPH^•^ is a stable free radical compound with a characteristic absorption at a wavelength of 517 nm. Antioxidants upon interaction with DPPH^•^ either transfer an electron or hydrogen atom to DPPH^•^, thus neutralizing its free radical character. The colour of the reaction mixture changes from purple to yellow resulting in an absorbance decrease. The degree of discolouration indicates the scavenging potential of the antioxidants [47,48]. As shown in Table 1, all the tested samples demonstrated DPPH^•^ scavenging activity in a concentration dependant manner, with a wide range of IC_50_ values from 3.08 µg/mL to 43.97 µg/mL. The ethanolic extract of *Satureja subspicata* exhibited the most effective radical scavenging ability, followed by *Origanum vulgare* (4.19 µg/mL) and *Salvia officinalis* (4.81 µg/mL). Some other tested plants, such as *Rosmarinus officinalis*, *Teucrium polium*, *T. chamaedrys*, *Micromeria thymifolia*, *Melissa officinalis*, *Teucrium montanum*, *Mentha longifolia*, *M.* *x piperita*, *Micromeria graeca* and *Satureja montana*, also showed potent antioxidant activity (IC_50_ = 5.06–9.95 µg/mL). Caffeic, rosmarinic, and chlorogenic acids with IC_50_ values of 0.36, 1.01 and 1.69 μg/mL, respectively, demonstrated the strongest antioxidative properties, even significantly stronger than trolox (IC_50_ = 1.99 µg/mL), while ferulic acid was a weaker free radical scavenger (IC_50_ = 3.56 µg/mL). 

The total antioxidant capacities of Lamiaceae ethanolic extracts in comparison with hydroxycinnamic acids were evaluated using the phosphomolybdenum method. This assay is based on the reduction of Mo(VI) to Mo(V) by the antioxidant compounds and the subsequent formation of a green phosphate/Mo(V) complex at acidic pH with a maximal absorption at 695 nm [48]. The obtained results, expressed as trolox equivalents (TE), are presented in Table 1. All investigated samples were active in a concentration dependent manner. Among investigated plants, *Melissa officinalis*, *Salvia officinalis*, *Mentha longifolia*, *Origanum vulgare*, *Teucrium montanum*, *Mentha x piperita*, *Teucrium chamaedrys* and *Satureja montana* possessed the highest total antioxidant capacities ranging between 717.41 mg TE/g and 855.78 mg TE/g. Hydroxycinnamic acids were much more active than tested extracts with values of 1240.65–1940.38 mg TE/g. Their potency decreased in the following order: caffeic acid > ferulic acid > rosmarinic acid > chlorogenic acid. Our results are in agreement with previous research on hydroxicinnamic acids [49,50,51] and suggest their great possible influence on antioxidant effects of the tested Lamiaceae extracts.

**Table 1:** Antioxidant activities of the ethanolic extracts of selected Lamiaceae species and their phenolic acid constituents.

Sample	Voucher number (FGHR)	Plant part used	Extract yield ^a^	DPPH radical scavenging activity	Total antioxidant capacity (mg TE/g)
				IC_50_ (μg/mL)	
**Medicinal plant**					
*Acinos arvensis* (Lam.) Dandy	L0111	Aerial part	15.47	17.50 ± 0.42	254.97 ± 2.68
*Calamintha grandiflora* (L.) Moench	L0141	Aerial part	5.80	10.75 ± 0.22	609.03 ± 0.38
*Calamintha officinalis* Moench	L0142	Aerial part	9.02	16.45 ± 0.32	650.65 ± 3.44
*Calamintha sylvatica* Bromf.	L0143	Aerial part	11.62	18.61 ± 0.50	449.84 ± 3.82
*Clinopodium vulgare* L.	L0151	Aerial part	7.98	17.39 ± 0.13	473.62 ± 1.53
*Lavandula angustifolia* Mill.	L1101	Flower	11.05	31.62 ± 0.84	577.95 ± 13.76
*Lavandula x intermedia* Emeric ex Loisel.	L1102	Flower	16.86	43.97 ± 0.47	439.03 ± 15.29
*Marrubium incanum* Desr.	L1141	Aerial part	4.31	11.82 ± 0.33	502.54 ± 1.15
*Melissa officinalis* L.	L1151	Leaf	5.79	7.74 ± 0.01	855.78 ± 6.12
*Mentha longifolia* (L.) Huds.	L1173	Aerial part	7.34	8.43 ± 0.28	830.38 ± 6.12
*Mentha pulegium* L.	L1176	Aerial part	7.52	24.27 ± 0.21	518.49 ± 22.17
*Mentha x piperita* L.	L1175	Leaf	5.62	8.88 ± 0.13	790.38 ± 19.11
*Micromeria croatica* (Pers.) Schott	L1181	Aerial part	7.24	29.48 ± 0.51	530.11 ± 26.37
*Micromeria graeca* (L.) Benth. ex Reich.	L1184	Aerial part	7.42	8.79 ± 0.01	580.11 ± 3.06
*Micromeria juliana* (L.) Benth. ex Rchb.	L1185	Aerial part	4.69	17.52 ± 0.33	403.62 ± 21.79
*Micromeria thymifolia* (Scop.) Fritsch	L1189	Aerial part	6.67	6.53 ± 0.13	478.49 ± 7.64
*Origanum vulgare* L.	L1202	Aerial part	13.47	4.19 ± 0.04	803.89 ± 0.76
*Rosmarinus officinalis* L.	L1241	Leaf	19.32	5.06 ± 0.01	602.00 ± 3.44
*Salvia officinalis* L.	L1259	Leaf	12.40	4.81 ± 0.30	838.22 ± 12.61
*Satureja montana* L.	L1263	Aerial part	7.39	9.95 ± 0.06	717.41 ± 1.53
*Satureja subspicata* Bartl. ex Vis.	L1264	Aerial part	17.60	3.08 ± 0.04	406.59 ± 19.11
*Teucrium arduini* L.	L1301	Aerial part	12.48	18.06 ± 0.68	530.11 ± 30.96
*Teucrium chamaedrys* L.	L1304	Aerial part	11.34	5.97 ± 0.11	792.81 ± 11.85
*Teucrium montanum* L.	L1308	Aerial part	14.44	7.78 ± 0.13	771.19 ± 8.03
*Teucrium polium* L.	L1309	Aerial part	15.67	5.90 ± 0.12	589.30 ± 14.52
*Thymus vulgaris* L.	L1318	Aerial part	7.25	12.27 ± 0.07	679.83 ± 12.96
**Phenolic acid constituent**					
Rosmarinic acid				1.01 ± 0.07	1894.84 ± 10.51
Chlorogenic acid				1.69 ± 0.02	1240.65 ± 59.24
Caffeic acid				0.36 ± 0.03	1940.38 ± 5.35
Ferulic acid				3.56 ± 0.36	1923.62 ± 0.20
Trolox (referent antioxidant)				1.99 ± 0.03	-

^a^ Extract yield expressed as percentage weight of air-dried plant material.

### 2.3. Content and Composition of Hydroxycinnamic Acids

The contents of total hydroxycinnamic derivatives in the ethanolic extracts of selected Lamiaceae species were determined spectrophotometrically and expressed as mg rosmarinic acid per g of dry plant extract (Table 2). The presented results revealed that the extracts differed greatly in hydroxycinnamic acids content, ranging from 10.75 mg/g to 173.10 mg/g. *Satureja subspicata* contained the highest amount of these polyphenolic compounds, whereas their lowest level was found in *Teucrium arduini*. In addition to *Satureja subspicata*, the extracts of *Mentha* *x* *piperita*, *Origanum vulgare*, *Satureja* *montana*, *Salvia officinalis*, *Micromeria graeca*, *Mentha longifolia*, *Micromeria thymifolia*, *Melissa officinalis*, *Calamintha grandiflora* and *Thymus vulgaris* were also characterized by a high hydroxycinnamic acids content (72.98–157.45 mg/g). 

**Table 2:** Content and composition of hydroxycinnamic derivatives of Lamiaceae ethanolic extracts.

Medicinal plant	Plant part used	Total hydroxycinnamic derivatives ^a^	Rosmarinic acid ^b^	Chlorogenic acid ^b^	Caffeic acid ^b^	Ferulic acid ^b^
*Acinos arvensis*	Aerial part	36.58 ± 0.42	2.21	26.06	3.53	nd
*Calamintha grandiflora*	Aerial part	75.42 ± 0.74	24.08	2.01	0.37	nd
*Calamintha officinalis*	Aerial part	30.17 ± 0.44	3.82	6.88	0.57	nd
*Calamintha sylvatica*	Aerial part	31.65 ± 0.19	0.70	11.18	0.74	nd
*Clinopodium vulgare*	Aerial part	27.12 ± 0.71	12.27	2.25	0.56	nd
*Lavandula angustifolia*	Flower	42.64 ± 0.19	3.31	3.22	nd	1.31
*Lavandula x intermedia*	Flower	26.00 ± 0.38	3.04	1.00	0.33	0.11
*Marrubium incanum*	Aerial part	57.78 ± 1.25	5.01	1.14	0.47	nd
*Melissa officinalis*	Leaf	77.70 ± 0. 94	39.48	1.16	1.05	nd
*Mentha longifolia*	Aerial part	80.06 ± 1.76	22.33	1.50	1.18	nd
*Mentha pulegium*	Aerial part	34.83 ± 0.38	12.72	1.64	0.72	nd
*Mentha x piperita*	Leaf	157.45 ± 2.71	61.05	1.78	2.15	0.50
*Micromeria croatica*	Aerial part	30.13 ± 0.33	9.95	1.26	0.70	nd
*Micromeria graeca*	Aerial part	81.76 ± 0.76	18.41	1.26	0.79	0.27
*Micromeria juliana*	Aerial part	41.50 ± 0.25	21.62	1.29	0.91	0.28
*Micromeria thymifolia*	Aerial part	79.86 ± 1.51	26.50	13.22	2.98	0.93
*Origanum vulgare*	Aerial part	129.16 ± 0.76	37.73	nd	1.12	nd
*Rosmarinus officinalis*	Leaf	55.57 ± 0.47	18.94	1.18	0.63	nd
*Salvia officinalis*	Leaf	86.86 ± 0.59	18.72	1.13	0.80	0.77
*Satureja montana*	Aerial part	95.29 ± 0.66	31.11	1.19	0.65	nd
*Satureja subspicata*	Aerial part	173.10 ± 1.68	46.11	1.62	1.78	nd
*Teucrium arduini*	Aerial part	10.75 ± 0.72	nd	1.75	nd	0.97
*Teucrium chamaedrys*	Aerial part	18.58 ± 0.97	1.03	4.06	nd	1.37
*Teucrium montanum*	Aerial part	15.11 ± 1.67	nd	2.25	2.36	1.70
*Teucrium polium*	Aerial part	13.91 ± 0.42	nd	2.19	2.36	1.38
*Thymus vulgaris*	Aerial part	72.98 ± 0.18	32.80	1.18	2.07	0.49

^a^ Content determined spectrophotometrically and expressed as mg rosmarinic acid/g of extract; ^b^ Content determined by HPLC and expressed as mg/g of extract; nd – not detected.

**Figure 2:**
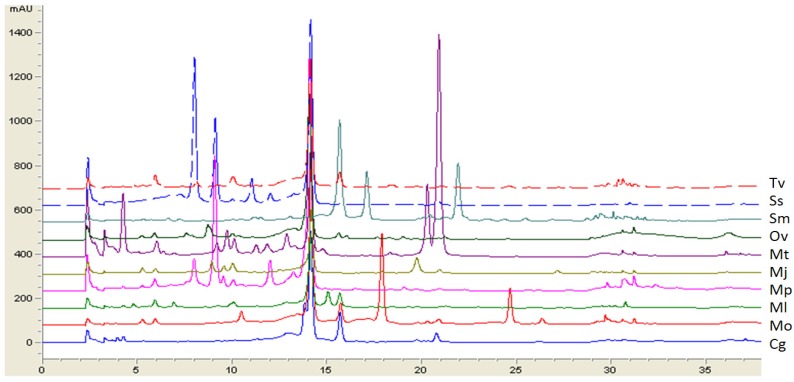
Overlay of HPLC-DAD chromatograms at 330 nm of the ethanolic extracts of Lamiaceae medicinal plants rich in rosmarinic acid (Rt = 14.1 min).

Abbreviations: Cg - *Calamintha grandiflora*, Mo - *Melissa officinalis*, Ml - *Mentha longifolia*, Mp - *Mentha x piperita*, Mj - *Micromeria juliana*, Mt - *Micromeria thymifolia*, Ov - *Origanum vulgare*, Sm - *Satureja montana*, Ss - *Satureja subspicata*, Tv - *Thymus vulgaris*.

Furthermore, RP-HPLC analyses of rosmarinic, chlorogenic, caffeic and ferulic acids, as the most common free hydroxycinnamic acids present in Lamiaceae species [11,52,53], were carried out and the obtained results are presented in Table 2. The main components of most of the tested extracts were rosmarinic (0.70–61.05 mg/g) and chlorogenic acids (1.00–26.06 mg/g), while caffeic (0.33–3.53 mg/g) and ferulic acids (0.11–1.70 mg/g) were generally less abundant. Figure 2 shows the respective HPLC profiles of ten ethanolic extracts containing more than 20 mg/g of rosmarinic acid found to be the predominant constituent of most of the investigated Lamiaceae species. These results highlight *Mentha* *x* *piperita*, *Satureja subspicata* and *Melissa officinalis* as rich sources of rosmarinic acid, while *Acinos arvenis* had a high content of chlorogenic acid (26.06 mg/g).

The obtained results showed a very strong positive correlation between rosmarinic acid and total hydroxycinnamic acids content (*r* = 0.905, *p* < 0.001), indicating the great contribution of rosmarinic acid to the content of total hydroxycinnamic acids in Lamiaceae species. Comparing the total hydroxycinnamic acids content with the chlorogenic, caffeic and ferulic acid contents, no significant correlations were observed (*r* values range from −0.313 to 0.234 with *p* = 0.119–0.353). The influence of rosmarinic acid content on AChE inhibitory and antioxidant properties of the tested Lamiaceae species was also evaluated. No significant correlation was found between rosmarinic acid content and AChE inhibitory activity at 1 mg/mL (*r* = 0.150, *p* = 0.465). However, a moderate positive correlation was observed (*r* = 0.533, *p* = 0.011) between rosmarinic acid content and anti-AChE activity (1 mg/mL) for the investigated plants, excluding *Teucrium* species. The correlation between rosmarinic acid content and DPPH activity for all plant extracts was moderate (*r* = −0.441, *p* = 0.024), and even strong for plant extracts excluding *Teucrium* species (*r* = −0.614, *p* = 0.002). There was no statistically significant correlation between rosmarinic acid content and total antioxidant capacity (*r* = 0.344, *p* = 0.086), while a moderate correlation was observed in comparing the investigated Lamiaceae species excluding *Teucrium* species (*r* = 0.507, *p* = 0.016). These results are consistent with previously published data [30,31,32,54], and therefore, rosmarinic acid is highly likely to contribute substantially to AChE inhibitory and antioxidant activities of most studied Lamiaceae species. However, the notable activity of tested *Teucrium* species containing low amounts of hydroxycinnamic acids could be attributed to their terpenic components which are well-known to possess anti-AChE properties [42]. On the other hand, their significant antioxidant properties likely depend on flavonoids and other present phenolic compounds [55]. The findings suggest that Lamiaceae extracts that contain both terpenes and polyphenols could affect different targets, including AChE activity and oxidative stress.

## 3. Experimental

### 3.1. Plant Material and Extraction Procedure

A total of 26 wild-growing Lamiaceae species used in this study (Table 1) were collected before (leaves) and during the flowering period (aerial parts and flowers), between May and September 2011, in Croatia. The plant material was identified at the Department of Pharmacognosy, Faculty of Pharmacy and Biochemistry, University of Zagreb, Croatia, where the voucher specimens have been deposited. Air-dried and pulverized plant material (5.00 g) was extracted with ethanol (50 mL) for 30 min using an ultrasonic bath. The extract was then filtered through Whatman No. 1 paper and concentrated to dryness under a vacuum on a rotary evaporator at 50 °C. The extraction yields are given in Table 1.

### 3.2. Chemicals

Acetylcholinesterase (AChE) from electric eel (Type-VI-S, EC 3.1.1.7), acetylthiocholine iodide (ATChI), ammonium molybdate, chlorogenic acid (≥95%), 2,2-diphenyl-1-picryl-hydrazyl (DPPH^•^), 5,5'-dithiobis(2-nitrobenzoic acid) (DTNB), galanthamine hydrobromide, sodium molybdate, sodium nitrite, rosmarinic acid (96%) and Trizma base were obtained from Sigma-Aldrich (St. Louis, MO, USA). Sodium hydroxide and sodium phosphate were purchased from Kemika (Zagreb, Croatia). Caffeic acid (≥95%), ferulic acid (≥98%) and trolox (≥98%) were obtained from Fluka (Buchs, Switzerland). All other chemicals and reagents used were of analytical grade.

### 3.3. Acetylcholinesterase (AChE) Inhibition Assay

The AChE inhibitory activities of the Lamiaceae extracts and their phenolic acid constituents were determined using modified Ellman’s colorimetric method described by Conforti *et al.* [56]. In brief, 1900 μL of 50 mM Tris-HCl buffer (pH 8.0), 40 μL of 0.02 U/mL AChE and 20 μL of tested solution (0.25–1 mg/mL) were mixed and pre-incubated for 30 min at 4 °C. The reaction was then initiated with the addition of 20 μL of 10 mM DTNB and 20 μL of 12 mM ATChI. AChE activity was determined spectrophotometrically through measuring the change in ultraviolet absorbance of an assay solution at 412 nm over a period of 10  min at 25 °C. Galanthamine (0.03–1 μg/mL) was used as positive control. Percentage enzyme inhibition was calculated by comparing the enzymatic activity with, and without inhibitor. The experiment was run in triplicate.

### 3.4. DPPH Free Radical Scavenging Assay

The free radical scavenging activities of the investigated Lamiaceae extracts and their phenolic acid constituents were evaluated using the stable DPPH radicals according to the previously reported method [57]. Briefly, 0.1 mM solution of DPPH in ethanol was prepared and 1 mL of this solution was added to 3 mL of the ethanolic sample solution. The samples were assayed in a range of 0.2–100 μg/mL. After incubation for 30 min in the dark at room temperature, the absorbance was measured spectrophotometrically at 517 nm. Trolox was used as the reference antioxidant compound. The assay was carried out in triplicate. The capability to scavenge the DPPH radicals was calculated using the following equation: (%) = (1 − A_1_/A_0_) × 100, where A_0_ is the absorbance of the control reaction and A_1_ is the absorbance in the presence of sample, corrected for the absorbance of sample itself. The concentrations of samples that provide 50% inhibition (IC_50_) were obtained by interpolation from linear regression analysis.

### 3.5. Total Antioxidant Capacity Assay

The total antioxidant capacities of the studied Lamiaceae plant extracts and their phenolic acid constituents were evaluated by the phosphomolybdenum method previously described by Vladimir-Knežević *et al.* [48]. An aliquot of sample solution in ethanol (0.3 mL, 50 and 100 μg/mL) was combined in a vial with reagent solution (2.7 mL) containing 0.6 M sulfuric acid, 28 mM sodium phosphate and 4 mM ammonium molybdate. The reaction mixture was incubated at 95 °C for 90 min. After cooling to room temperature, the absorbance was measured spectrophotometrically at 695 nm. The assay was run in triplicate. The antioxidant capacity of the sample was expressed as trolox equivalents (TE), utilizing a calibration curve of trolox. 

### 3.6. Determination of Total Hydroxycinnamic Derivatives

For the determination of total hydroxycinnamic acids content, the following procedure described in European Pharmacopoeia was performed [58]. Briefly, the plant extract (0.200 g) was mixed with 50% ethanol (80 mL) and boiled in a water bath under a reflux condenser for 30 min. The cooled extract was filtered into a 100 mL volumetric flask, the filter rinsed, and diluted to 100.0 mL with 50% ethanol. The obtained solution served as stock solution. Test solution was prepared by mixing stock solution (1.0 mL) with 0.5 M hydrochloric acid (2 mL), 2 mL of a solution prepared by dissolving 10 g of sodium nitrite and 10 g of sodium molybdate in 100 mL of water, dilute sodium hydroxide (2 mL, 8.5%) and diluted to 10.0 mL with water. The absorbance of the test solution was immediately measured spectrophotometrically at 505 nm, using a compensation solution prepared by diluting 1.0 mL of the stock solution. Each sample was tested in triplicate and the content of total hydroxycinnamic derivatives was expressed as mg of rosmarinic acid per g of dry plant extract. 

### 3.7. HPLC Analysis

The hydroxycinnamic acids composition of the selected Lamiaceae species was studied using a slightly modified method of Fecka and Turek [59]. HPLC analysis was conducted on an Agilent 1100 Series instrument (Agilent Technologies, Santa Clara, CA, USA) equipped with an Agilent auto sampler, a quaternary pump, a column thermostat, and a photodiode array detector. The separation was achieved using a LichroCART^®^250-4 column (250 mm × 4.0 mm i.d.) packed with LiChrospher^®^ 100 RP-18e (5 μm). The mobile phase consisted of acidified acetonitrile (5% formic acid in acetonitrile, solvent A) and acidified water (5% formic acid in water, solvent B). The gradient employed was as follows: 0 min, 15% A; 25 min, 35% A; 27 min, 70% A; 32 min, 70% A; 33 min, 100% A; 38 min, 100% A. The flow rate was 0.9 mL/min. The injection volume for the sample was 20 μL. All chromatographic experiments were performed at 20 °C. Polyphenolic profiles of plant extracts were recorded at a wavelength of 330 nm. Chromatographic data were acquired and processed using HP ChemStation software. Phenolic acids were identified based on their chromatographic retention times and UV spectra, and quantified by comparing integrated peak areas to calibration curves prepared with corresponding analytical standards. The results were expressed in mg per g of the dry extract. All HPLC analyses were run in duplicate.

### 3.8. Statistical Analysis

The data analysis was performed using Microsoft Excel 2007 software and MedCalc for Windows (version 12.5.0.0.). The results were tested for normal distribution by Kolmogorov-Smirnov test. Correlations were assessed using Pearson’s correlation coefficient (*r*) and *p* < 0.05 was considered statistically significant. 

## 4. Conclusions

This paper presents the results of a comparative study of AChE inhibitory and antioxidant activities of a large number of Lamiaceae medicinal plants, providing the first-ever data for most of them. Of the 26 tested ethanolic extracts, eight did not achieve 50% inhibition of AChE at a concentration of 1 mg/mL, whereas nine extracts showed strong inhibitory activity, achieving values over 75%. These findings suggest the great influence of the rosmarinic acid content and total hydroxycinnamic derivatives on the AChE inhibitory properties of tested plants, with the exception of *Teucrium* species, whose exceptional effects on AChE could be associated with other constituents such as terpenes. All the tested extracts also demonstrated moderate to strong antioxidant activities, primarily due to their high levels of polyphenols. The obtained results showed that Lamiaceae species containing multifunctional phenolic and terpenic components that can affect different targets, such as AChE activity and oxidative stress, may have great relevance in the prevention and therapy of AD and other neurodegenerative disorders.
